# Low Driveline Infection Rates in Patients With a Novel Fully Magnetically Levitated Ventricular Assist Device

**DOI:** 10.1093/ejcts/ezaf365

**Published:** 2025-10-25

**Authors:** Xianqiang Wang, Xingtong Zhou, Haibo Chen, Haotian Zhang, Yanming Zhang, Yong Cui, Keli Huang, Zhengdong Hua, Yang Yan, Mi Zhou, Qiang Zhao, Xiaoning Sun, Chunsheng Wang, Zhenwei Ge, Aiqiang Dong, Shengshou Hu

**Affiliations:** Centre of Cardiac Surgery in Adults, Fuwai Hospital, National Center for Cardiovascular Diseases, Chinese Academy of Medical Sciences and Peking Union Medical College, Beijing, 100037, China; Centre of Cardiac Surgery in Adults, Fuwai Hospital, National Center for Cardiovascular Diseases, Chinese Academy of Medical Sciences and Peking Union Medical College, Beijing, 100037, China; Centre of Cardiac Surgery in Adults, Fuwai Hospital, National Center for Cardiovascular Diseases, Chinese Academy of Medical Sciences and Peking Union Medical College, Beijing, 100037, China; Centre of Cardiac Surgery in Adults, Fuwai Hospital, National Center for Cardiovascular Diseases, Chinese Academy of Medical Sciences and Peking Union Medical College, Beijing, 100037, China; Centre of Cardiac Surgery in Adults, Fuwai Hospital, National Center for Cardiovascular Diseases, Chinese Academy of Medical Sciences and Peking Union Medical College, Beijing, 100037, China; Department of Cardiothoracic Surgery, Zhejiang Provincial People’s Hospital, Hangzhou, 310014, China; Department of Cardiac Surgery, Sichuan Provincial People’s Hospital, Chengdu, 610072, China; Department of Cardiac Surgery, Asia Heart Hospital, Wuhan, 430022, China; Department of Cardiovascular Surgery, The First Affiliated Hospital of Xi’an Jiaotong University, Xi’an, 710061, China; Department of Cardiovascular Surgery, Ruijin Hospital, Shanghai Jiaotong University School of Medicine, Shanghai, 200025, China; Department of Cardiovascular Surgery, Ruijin Hospital, Shanghai Jiaotong University School of Medicine, Shanghai, 200025, China; Department of Cardiac Surgery, Zhongshan Hospital, Fudan University, Shanghai, 200032, China; Department of Cardiac Surgery, Zhongshan Hospital, Fudan University, Shanghai, 200032, China; Department of Cardiovascular Surgery, Henan Provincial Chest Hospital, Zhengzhou, 450003, China; Department of Cardiovascular Surgery, Zhejiang University College of Medicine Second Affiliated Hospital, Hangzhou, 310009, China; Centre of Cardiac Surgery in Adults, Fuwai Hospital, National Center for Cardiovascular Diseases, Chinese Academy of Medical Sciences and Peking Union Medical College, Beijing, 100037, China

**Keywords:** driveline infection, advanced heart failure, left ventricular assist device, CH-VAD

## Abstract

**Objectives:**

Driveline infection remains a major complication of long-term ventricular assist device therapy. Previous studies have associated the risk of infection with driveline design and physical properties. The CH-VAD is a fully magnetically levitated pump with an ultrathin (diameter 3.3 mm), highly flexible driveline designed to reduce infection risk. This study was conducted to assess the incidence, risk factors, and approaches to prevention and management of driveline infection in CH-VAD patients (NCT06878456).

**Methods:**

All patients who underwent CH-VAD implantation between June 2017 and October 2024 at 9 centres were retrospectively analysed. Data related to clinical characteristics, surgical procedures, and driveline infection prevention and management details were collected, and risk factors were analysed.

**Results:**

A total of 181 consecutive patients were included, with a median age of 56 years and 86% male. During a median support duration of 1.6 years, driveline infections occurred in 20 out of 181 patients (11.0%). Twenty-six events were observed, corresponding to a rate of 0.07 events per patient-year. Cumulative incidence of the first driveline infection was 2.8% at 6 months, 5.6% at 12 months, and 8.5% at 24 months. Frequent dressing changes were found to be significantly associated with increased driveline infection risk (HR 3.773, *P* = .022).

**Conclusions:**

Patients treated with CH-VADs experienced an encouraging low rate of driveline infection, considered among the lowest reported for durable ventricular assist devices. These findings have implications for both clinical management and driveline design.

## Graphical abstract

**Figure:**
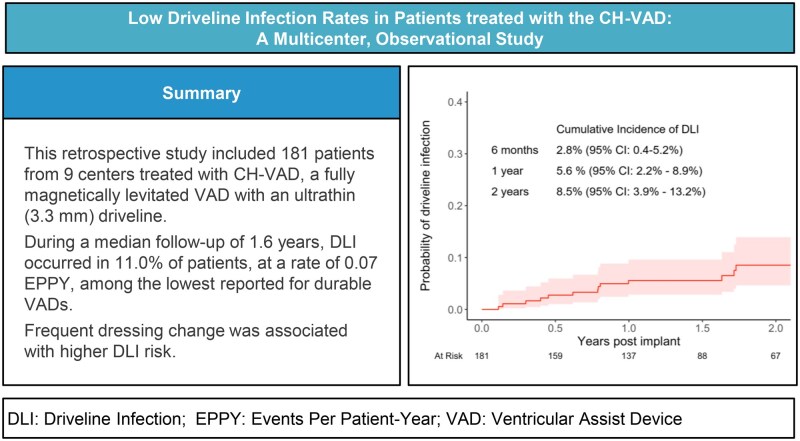

## INTRODUCTION

Heart failure is a major cause of morbidity and mortality worldwide due to ageing populations and improved survival rates after cardiovascular conditions.^1^ Patients with advanced heart failure may require mechanical circulatory support, such as left ventricular assist devices (LVADs). Despite significant advances in LVAD technology and subsequent improvements in outcomes, driveline infection (DLI) remains one of the major complications in this therapy, significantly impairing patient’s quality of life and prognosis.^2^

Over the years, a variety of LVADs with different designs have been introduced into clinical practice. The risks contributing to DLI are similar across devices, including trauma to the exit site from external or internal forces, and any condition that puts the exit site at risk for microbial invasion. Evidence suggests that the mechanical driveline features are modifiable risk factors for DLI.^3^

The CH-VAD (BrioHealth Technologies, Suzhou, China) is a centrifugal fully magnetically levitated LVAD with advanced driveline features. Compared with existing devices such as HeartMate 3, CH-VAD has a smaller (3.3 mm vs 6.6 mm)^3^ and more flexible driveline,^4^ which aims to reduce local trauma at the exit site and potentially lower infection risk. The device was first implanted in China in June 2017. Following evidence from compassionate use and a prospective, multicentre, single-arm trial, the device was approved by the Chinese National Medical Products Administration for commercial use in 2021.

A previous single-centre experience with 50 CH-VAD patients reported favourable survival and low rates of adverse events.^5^ This current multicentre, retrospective, observational study aims to evaluate DLI incidence in CH-VAD patients across 9 centres in China and to explore underlying factors that may contribute to DLI.

## PATIENTS AND METHODS

### Ethics statement

This study was conducted in accordance with the Declaration of Helsinki and approved by the Institutional Review Board of Fuwai Hospital on November 6th, 2023 (Approval number: 2023-2104). Patient data were deidentified, and the requirement for informed consent was waived. This study was registered on ClinicalTrials.gov (NCT06878456).

### Device description

**Figure 1.**
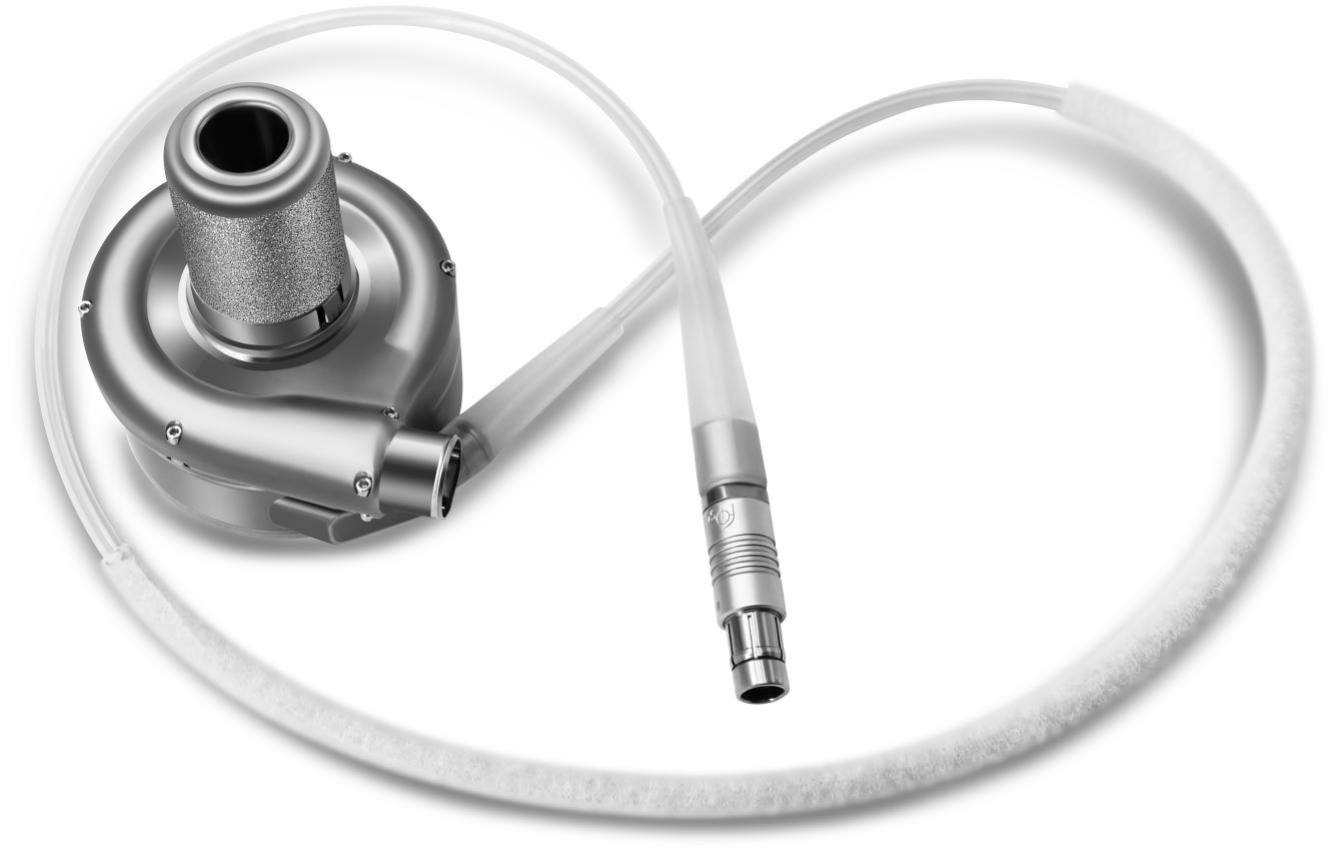
The CH-VAD Pump and the Driveline

The CH-VAD left ventricular assist system (LVAS) is designed to enhance haemocompatibility while reducing surgical invasiveness and infection risk. Its main components include the blood pump, outflow graft, the driveline, an external controller, and portable batteries. The implanted pump and driveline are shown in **Figure 1**.

The CH-VAD pump utilizes a fully magnetically levitated rotor, with a magnetic bearing system mechanically decoupled from the electric motor. This design permits the integration of a larger rotor within a compact housing. The internal flow path has been engineered to optimize haemodynamics, promoting efficient blood flow while minimizing regions of turbulence and flow stagnation.^6^

**Figure 2.**
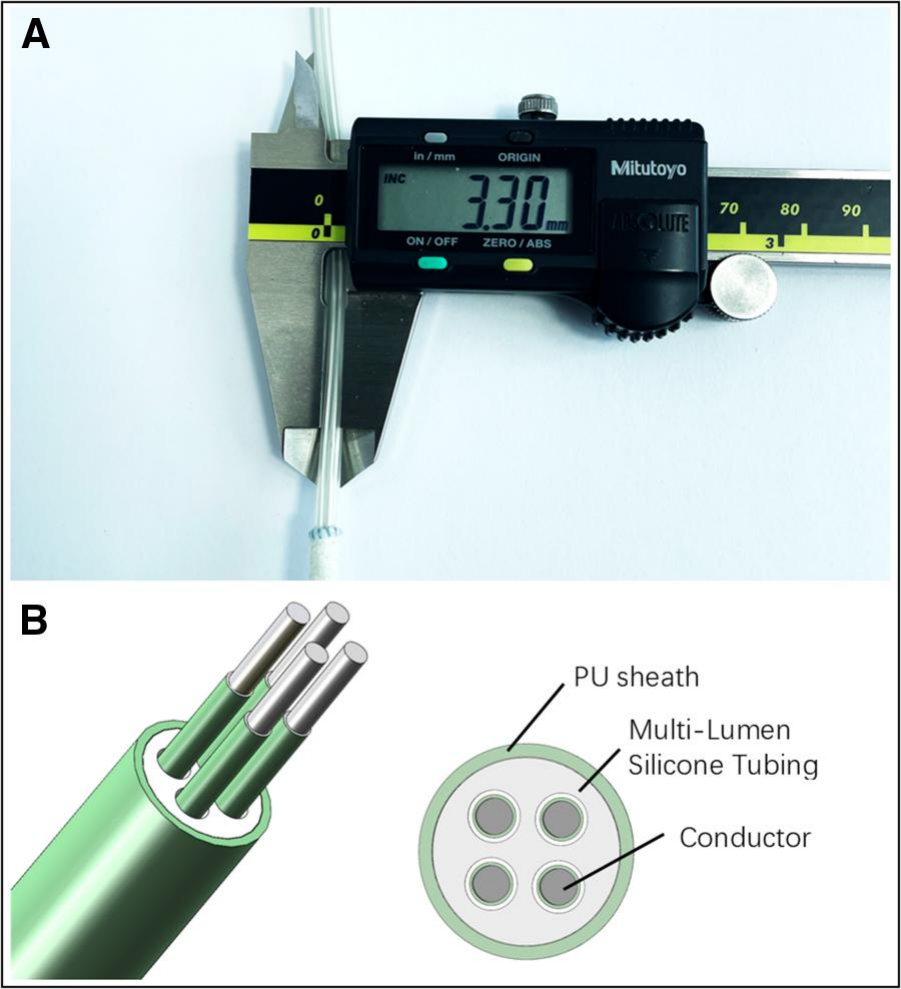
External view and internal structure of the CH-VAD driveline. (A) Close-up view of the CH-VAD driveline showing the external diameter. (B) The internal structure of the driveline

The CH-VAD LVAS is equipped with an ultrathin driveline measuring 3.3 mm in diameter (**Figure 2A**), incorporating 4 conductors. This configuration minimizes driveline bulk and enhances flexibility relative to comparable LVAD systems, aiming to improve patient comfort and reduce the risk of DLI.

Communication between the controller and the pump is transmitted through power lines employing a proprietary power line communication technology, which minimizes the number of communication wires. The 4 internal conductors are designed with full redundancy, which ensures continuous pump operation even in the event of any single conductor failure. The driveline is fabricated with an outer polyurethane sheath and a multi-lumen silicone core, providing resistance to mechanical stress and environmental exposure while maintaining flexibility (**Figure 2B**). The sheath is not resistant to iodine-based disinfectants and chlorhexidine is recommended for routine disinfection.

### Patient population

This multicentre, retrospective, observational study included all patients who received CH-VAD implantation between June 2017 and October 2024 from 9 centres in China. The decision for LVAD implantation was made based on established international criteria.^7^ Patients were followed for at least 6 months or until death, transplant or explant; the last follow-up date was July 31, 2025.

### Definitions of DLIs

DLI was defined as clinical manifestation of infection with positive drainage culture, or culture-negative but with clear clinical evidence of the need for antibiotic or surgical intervention.^8^^,^^9^ Superficial infection was defined as preserved epithelialization at the exit site, while deep infection involved disrupted epithelialization or radiographic evidence of infection along the driveline tract.^9^^,^^10^ All DLI events were graded according to the DESTINE staging proposal.^11^ Successful resolution of a DLI event was defined as complete healing of the driveline exit site, with no redness, swelling, warmth, pain, or exudate, and no requirement for antibiotics beyond prophylactic treatment.

### Recommended infection prevention protocol

The infection prevention protocol followed in this study is based on the manufacturer’s recommendations and aligns with international guidelines.^10^ In summary, prevention measures begin 2 days before CH-VAD implantation with daily body scrubbing using chlorhexidine. Broad-spectrum prophylactic antibiotics are administered intravenously on the day of surgery. Topical antimicrobial irrigation of the surgical field is recommended before sternal wound closure.

The preferred driveline exit is in the right upper abdominal quadrant, 2-3 fingers widths below the costal margin along the midclavicular line, with the driveline pointing towards the right axilla. The driveline tunnel passes through the rectus abdominis and posterior sheath into the chest cavity, maximizing length within the muscle and forming a “U” shape. The driveline velour should be buried under the skin, with its distal end positioned 1-2 cm beneath the skin exit.

Temporary fixation of the driveline exit with non-absorbable anchoring sutures is recommended and should be removed 4-6 weeks after wound healing. Dressings should be changed every 2-3 days using chlorhexidine for disinfection. The driveline needs to be effectively immobilized to avoid bending and twisting.

In this study, key infection prevention steps were investigated, including skin decolonization, use of prophylactic antibiotics, exit-site location, temporary fixation, and dressing methods.

### Patient follow-up

Patients and caregivers receive training on driveline dressing changes and wound care before discharge. After discharge, patients are required to document the wound condition and attend regular follow-up visits for inspection. Patients stay in contact with the hospital’s long-term management team, sending updates and photographs of their driveline exit sites.

### Statistical analysis

Continuous variables are presented as means (standard deviations) or medians (interquartile ranges [IQRs]), depending on normality assessed by the Kolmogorov-Smirnov test. To identify risk factors for DLI, univariate Cox proportional hazards models were applied using time to first DLI or censoring. Variables for the multivariable Cox model were selected based on both statistical significance in univariable analysis (*P* < .05) and clinical relevance (diabetes as a recognized risk factor for device-related infections). Schoenfeld residual tests were used to validate the proportional hazards assumption. Cumulative incidence functions were calculated by the Aalen-Johansen method. Multivariable competing risk analyses and multiple imputation were performed as sensitivity analysis. Death, heart transplantation, and device explantation were considered as competing events. In the Cox proportional hazards regression analyses, variables with >20% missingness were excluded. For variables with moderate missingness (>10%), multiple imputation (10 datasets, pooled with Rubin’s rules) was used. A P  < .05 is considered statistically significant. Analyses were performed using R 4.5.1.

## RESULTS

### Patient characteristics

A total of 181 patients were included. The median age was 55.8 years, median body mass index (BMI) was 22.8 kg/m^2^, and 86% were male. The primary aetiology of heart failure was dilated cardiomyopathy (61%), and 25% had diabetes mellitus. Ninety-four percent of the patients were discharged from hospital with the device in place. Detailed baseline characteristics and operative data are summarized in **Table 1**.

**Table 1.** Patient Characteristics

Characteristics and parameters	*N* = 181
Age, years	55.8 (45.2-64.7)
Male, number (%)	156 (86.1%)
Height, cm	171.0 (166.5-176.0)
Body weight, kg	67.0 (57.0-78.5)
Body mass index, kg/m^2^	22.8 (20.2-26.1)
Body surface area, m^2^	1.87 ± 0.22
Aetiologies, number (%)	
Dilated cardiomyopathy	110 (60.8%)
Ischaemic aetiology	49 (27.1%)
Valvular disease	16 (8.8%)
Others	3 (3.3%)
Medical history, number (%)	
Stroke	11 (5.5%)
Myocardial infarction	39 (21.8%)
Diabetes mellitus	45 (24.8%)
Hypertension	50 (28.3%)
Previous cardiac surgery	11 (6.0%)
Smoking history	81 (45.8%)
Left ventricular ejection fraction, %	24.9 ± 6.2
Creatinine, μmol/L	103.3 (81.0-131.0)
BUN, mmol/L	8.77 (7.2-11.5)
eGFR	62.81 (48.9-84.0)
Sodium, mmol/L	139.20 ± 4.45
Total bilirubin, μmol/L	19.9 (13.7-28.2)
INTERMAC profiles, number (%)	
1	16 (8.8%)
2	57 (31.5%)
3	83 (45.9%)
4-5	24 (13.3%)
Undefined	1 (0.5%)
Surgical approach, number (%)	
Median sternotomy	150 (82.8%)
Lateral thoracotomy	31 (17.1%)
Concomitant surgery, number (%)	116 (64.0%)
Valve repair/replacement	68 (58.6%)
Left atrial appendage clipping/resection	50 (43.1%)
Coronary bypass grafting	23 (19.8%)
Cardiopulmonary bypass time, min	97.0 (64.0-140.0)
Length of stay, days	30.0 (23.0-41.0)
ICU stay, days	12.0 (8.0-21.0)
Duration of support, days	585 (397-873)
Survival to discharge, number (%)	170 (93.9%)

Abbreviations: ICU: intensive care unit; INTERMACS: Interagency Registry for Mechanically Assisted Circulatory Support; BUN, blood urea nitrogen; eGFR, estimated glomerular filtration rate.

### Incidence and rate of DLI

The median support duration was 1.60 (IQR 1.08-2.39) years, with the maximum follow-up of 7.0 years. DLI occurred in 20 of the 181 patients (11.0%, 95% CI, 6.9%-16.5%). Cumulative incidence of the first DLI was 2.8% at 6 months (95% CI, 0.4%-5.2%), 5.6% at 12 months (95% CI, 2.2%-8.9%), and 8.5% at 24 months (95% CI, 3.9%-13.2%) (Fig**ure 3**). Among affected patients, the median time to the first event was 1.32 years (range, 41 days to 3.5 years). A total of 26 events were observed in 20 patients; all occurred after hospital discharge. The event rate was 0.07 per patient-year (EPPY). Across the 9 centres, the median DLI event rate was 0.054 EPPY (IQR 0.045-0.086), with no significant inter-centre differences observed. Incidence was significantly higher (P<.001) in patients implanted before 2022 (27.3%, 9/33) compared to those implanted in 2022 or later (7.4%, 11/148).

**Figure 3.**
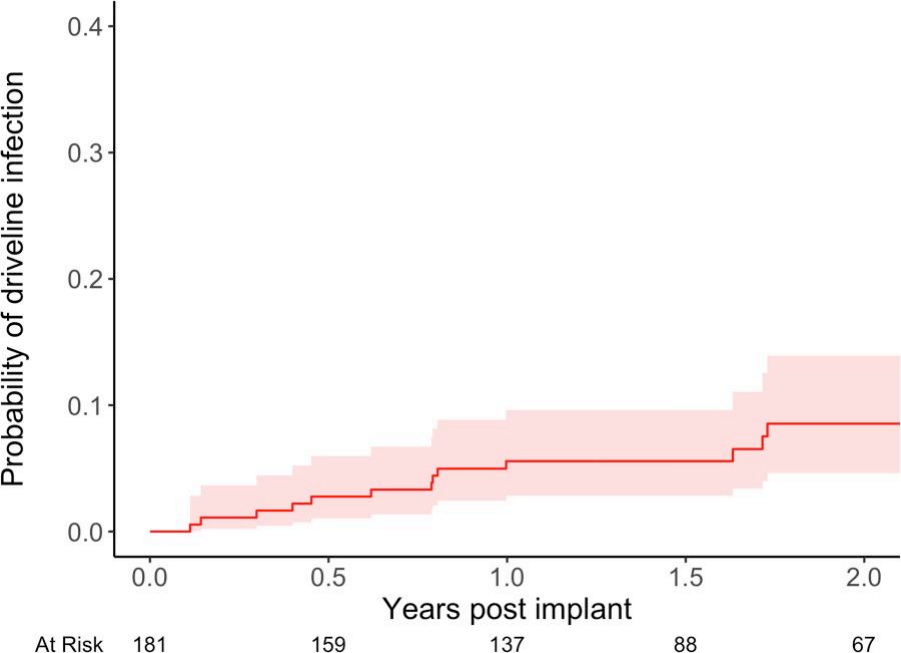
Cumulative Incidence of the First DLI

### Staging, treatment, and outcomes of DLIs

**Figure 4.**
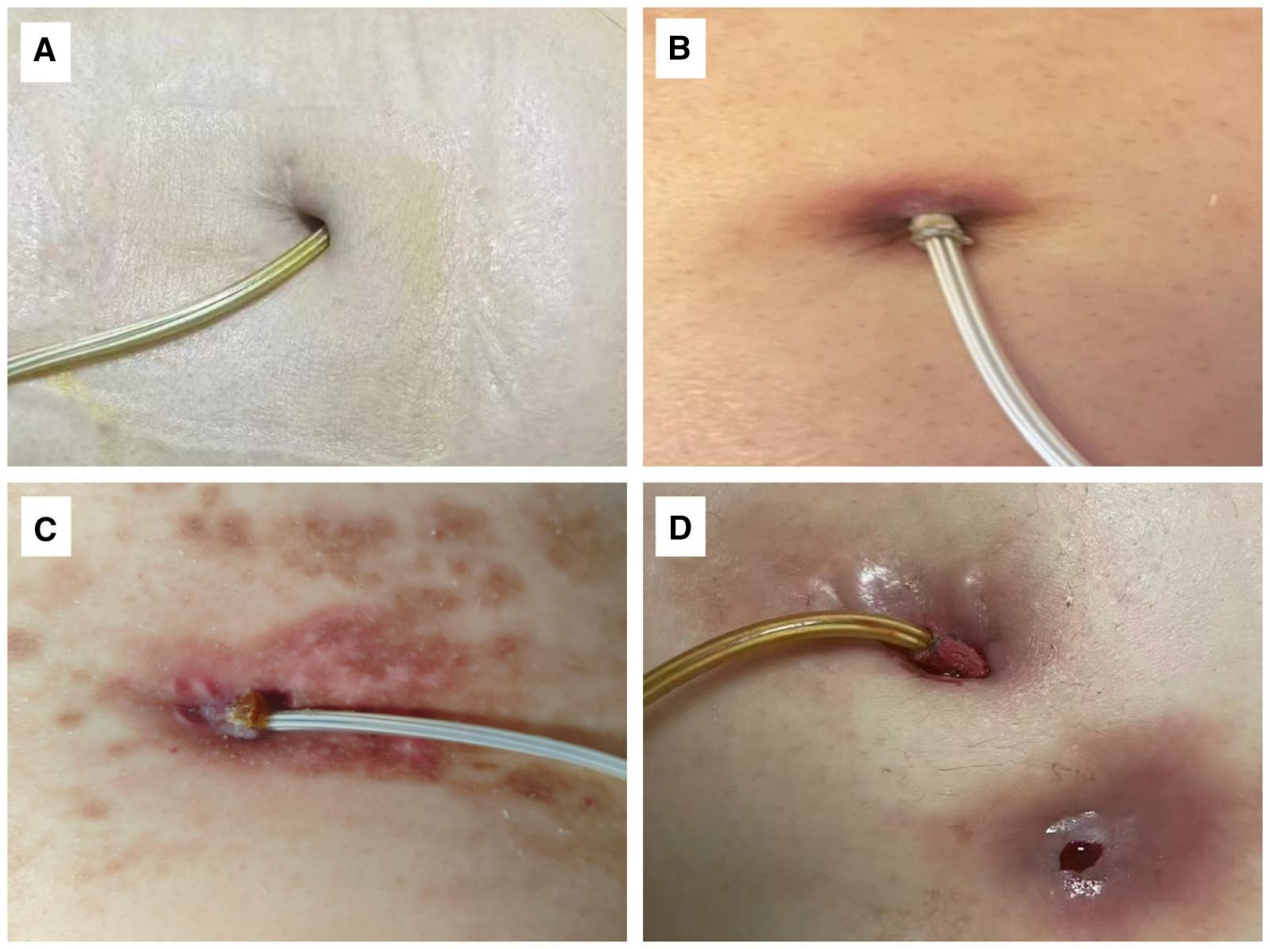
Representative examples of normal exit site and mild, moderate, and severe infections. (A) Normal driveline exit site. (B) Mild infection: local erythema without drainage. (C) Moderate infection: erythema with exudate and skin disruption. (D) Severe (deep) infection: subcutaneous fluid collection adjacent to the driveline exit with purulent or bloody discharge, indicating deep tissue involvement

According to the Interagency Registry for Mechanically Assisted Circulatory Support (INTERMACS) definition,^9^ 17 (65%) of the 26 events were classified as superficial infections, while 9 (35%) were considered deep infections. Staging according to The DESTINE proposal^11^ is shown in **Table 2**, and representative examples of mild, moderate, and severe infections are provided in **Figure 4**.

**Table 2.** Staging, Treatment Strategy, and Outcomes of DLIs

DESTINE stage, *number* (%)	*N* = 26
Stage 1—local wound healing disorder	
1a	0 (0.0%)
1b	4 (15.4%)
Stage 2—local infection	
2a	2 (7.7%)
2b	11 (42.3%)
Stage 3—systemic infection	5 (19.2%)
Stage 4—systemic infection with increased severity	3 (11.5%)
Stage 5—progressive systemic infection with deep DLI	1 (3.8%)
**Treatment strategy, *number* (%)**	***N* = 26**
Topical antibiotics	19 (73.1%)
Systematic antibiotics	15 (57.7%)
Surgical intervention	7 (26.9%)
VAC therapy	4 (15.4%)
Driveline relocation	1 (3.8%)
**Treatment outcomes, *number* (%)**	***N* = 26**
Successful resolution	23 (88.5%)
Persistent infection	2 (7.7%)
DLI-related death	1 (3.8%)

Abbreviations: DLI: driveline infection; VAC: vacuum-assisted closure.

Positive exudate cultures were identified in 20 out of 26 events. The most common pathogen was Staphylococcus aureus (65%). Additional blood cultures were collected in 6 events, of which 4 were positive, including 2 cases of *Staphylococcus aureus*, 1 case of *Streptococcus anginosus*, and 1 case of polymicrobial infection.

Management strategies varied depending on wound staging. For early signs such as redness or pain, dressing changes were adjusted to degree of wound wetness, and topical antibiotic ointments were applied in some centres. In cases with wound exudates, cultures and blood tests were conducted to guide empirical or targeted antibiotic use. Antibiotic therapy duration was typically 2-4 weeks when infections did not involve deep tissue and were extended to 2-3 months for deep infections after consultation with the infectious disease team. One patient with recurrent DLI required chronic suppressive antibiotic therapy. Irrigation with hydrogen peroxide or antibiotic solutions was used in deeper infections.

Among 20 patients with DLIs, 7 (35%) required surgical intervention, including incision and drainage or debridement. Four of them subsequently received vacuum-assisted closure (VAC) therapy, and 1 underwent driveline relocation, after which the wound healed successfully (**Figure S1**). No pump exchange or transplantation was needed.

Re-infection happened in 5 of 20 patients (25%). Three patients (3/20, 15%) had chronic infections, with 1 leading to mediastinitis and death. Treatment strategies and outcomes are summarized in **Table 2**.

### Infection prevention measures

All patients had perioperative prophylactic antibiotics as prescribed. Anti-*Staphylococcus aureus* agents, such as vancomycin or teicoplanin, were administered in 90% of cases, while cephalosporins were given to 82% of patients. Anti-fungal prophylaxis was included in 35% of the antibiotic regimens. Additionally, 33% of patients underwent antimicrobial irrigation of the surgical field before closure of the sternal wound. No significant association was observed between these measures and DLI risk.

The velour on the driveline was buried under the skin in 75% of patients. Patients with the buried DL had a significantly lower incidence of DLI (7.4%) compared to those with partial exposure (26.3%) (*P* < .001). The exit site location (left vs right), internal fixation, or temporary anchoring sutures showed no significant impact on DLI incidence.

Patients employed different methods to dress the exit site and secure the driveline (**Figure S2**). Most patients (82.4%) reported changing their dressings 2-5 times a week, while 9.5% changed once a week or less, and 8.1% more than 5 times per week. Chlorhexidine was used as disinfectant in 65% of patients. Six of the patients used silver ion dressings.

### Durability of the driveline

There were no driveline fractures or malfunctions during the observational period. In the early phase, 2 cases of sheath damage occurred after use of povidone-iodine as routine disinfectant. Both damages were repaired without affecting pump function and remain in normal use.

### Risk factor analysis

Baseline characteristics, surgical parameters, infection prevention, and driveline management measurements were analysed in the univariate regression analysis (**Table S1**). Four variables (age, height, diabetes, dressing change frequency) were included in the multivariate analysis. Results are presented in **Table 3**. The proportional hazards assumption was satisfied for all covariates (global Schoenfeld test *P* = .278) (**Figure S3**). Frequent dressing changes (>5 times per week vs 2-5 times per week) emerged as the only significant independent predictor of DLI, with a hazard ratio of 3.773 (95% CI, 1.210-11.762, *P* = .022).

**Table 3.** Cox Proportional Hazards Regression Analyses

	Univariate analyses		Multivariate analyses	
Variables	HR (95% CI)	*P*	HR (95% CI)	*P*
Age	0.962 (0.931, 0.995)	.023	0.976 (0.937-1.017)	.243
Height	1.069 (1.010, 1.132)	.022	1.023 (0.955-1.097)	.511
Diabetes	1.834 (0.729, 4.618)	.198	1.727 (0.628-4.749)	.290
Frequent dressing change^a^	5.239 (1.780, 15.424)	.003	3.773 (1.210-11.762)	.022

a >5 dressing changes per week vs 2-5 times per week.

Abbreviations: CI: confidence interval; HR: hazard ratio.

### Sensitivity analysis

In the competing risk analysis, frequent dressing changes remained significantly associated with increased DLI risk (sub-distribution HR 3.92, 95% CI, 1.13-13.60, *P* = .031). Multiple imputation analysis for variables with moderate missingness yielded consistent results (HR 4.56, 95% CI, 1.63-12.72, *P* = .004). Other covariates showed no statistically significant associations in both sensitivity analyses, consistent with the primary multivariable Cox regression results.

## DISCUSSION

This study provides the first comprehensive analysis of DLIs in CH-VAD patients, based on a relatively large cohort of 181 cases from both pre-market clinical trial and commercial use. Patients were enrolled over a 7-year period from 9 large medical centres across China. This representative patient population reflects the incidence, management and outcomes of DLIs in real-world practice of CH-VAD.

The application of full magnetic levitation technology in durable LVADs has substantially improved survival and reduced haemocompatibility-related adverse events.^12^ However, DLI remains one of the most challenging complications, with limited progress in recent years. Large-scale studies and registry consistently report DLI incidence ranging from 20% to over 50%.^13^ According to the INTERMACS report, the rate of device-related infections was 0.16-0.19 EPPY, most of which were DLIs.^14^ In this study, CH-VAD was associated with a low burden of DLI, with an event rate of 0.07 EPPY, among the lowest DLI rates reported in LVAD literature.

Standardized DLI prevention protocols are crucial for reducing infections. Previous studies have showed that keeping the driveline velour under the skin helps to prevent DLI,^15^^,^^16^ which is supported by our findings: patients with buried velour had a significantly lower DLI incidence than those with externalized velour (7.4% vs 26.3%).

We also found that patients implanted before 2022 had a significantly higher DLI incidence than those implanted more recently (27.3% vs 7.4%). In the early phase of clinical use, infection prevention was less standard, and both physicians and patients had limited experience in driveline care. Partial velour exposure was also more common in this period. It should also be noted that earlier patients had longer follow-up durations, which may have naturally increased cumulative infections. Therefore, extended studies are needed to confirm long-term trends. With continued refinement and standardization of driveline care, further reductions in DLIs with CH-VAD may be achievable.

Our multivariable analysis identified frequent driveline dressing change as a risk factor for DLI. This aligns with recent multicentre evidence from HeartMate 3 cohorts, where frequent dressing changes were associated with higher rates of late DLI, likely because repeated manipulation of the exit site increases opportunities for microbial entry and local trauma.^17^ These findings support a more judicious dressing change schedule once the driveline exit site has stabilized, while prospective studies are warranted to confirm causality.

Patient-related factors are also contributors to DLI risk. Obesity, prolonged hospital stays, diabetes mellitus, and other comorbidities are commonly reported as contributors.^13^ The median BMI of this cohort was 22.8, lower than data reported from Europe and the United States, which may have contributed to the lower infection rates.

In addition to management and patient-related factors, driveline design characteristics also play an important role in the development of DLI. Prior studies have shown that larger diameter and higher stiffness are associated with increased infection risk. For example, HeartMate II, with a smaller and more flexible driveline than EVAHEART and DuraHeart, was associated with lower DLI incidence over 2 years.^18^ Similarly, a meta-analysis and experimental study reported that the HeartMate3, with a thicker and stiffer driveline compared to HeartMate II and HVAD, had the highest 6-month DLI rate.^5^ CH-VAD has the thinnest driveline diameter (3.3 mm) compared to previous and current commercially available LVADs. A recent study compared the mechanical properties of 5 LVAD drivelines. BrioVAD, an updated version of CH-VAD that has the same driveline, was found to have the lowest bending stiffness and torque resistance.^4^ The ongoing INNOVATE trial (NCT02224755) in the United States is evaluating BrioVAD in comparison with HeartMate 3. This randomized controlled trial is expected to provide further evidence on this ultrathin driveline and enhance understanding in this field.

### Limitations

As a retrospective cohort study without a control group, it is subject to selection bias and residual confounding from unmeasured variables. The small number of DLI cases limited the statistical power to identify significant predictors. Some clinically relevant factors were not statistically confirmed, possibly due to low event rate or confounding effect of follow-up duration, as such management-related issues were more common in the earlier phase of clinical use. Finally, although the low incidence of DLI appeared strongly associated with CH-VAD driveline features, this relationship warrants further investigation.

## CONCLUSION

In this study, CH-VAD patients demonstrated a significant improvement in DLI outcomes, with an incidence considered among the lowest reported for durable LVADs. This improvement highlights the importance of not only careful clinical management but also advancements in driveline design.

## AUTHOR CONTRIBUTIONS

Xianqiang Wang (Conceptualization, Project administration, Data curation, Writing—original draft), Xingtong Zhou (Data curation, Methodology, Writing—original draft), Haibo Chen (Data curation, Methodology, Formal analysis, Writing—review & editing), Haotian Zhang (Statistic analysis, Writing—review & editing), Yanming Zhang (Data curation, Writing—review & editing), Yong Cui (Data curation, Writing—review & editing), Keli Huang (Data curation, Writing—review & editing), Zhengdong Hua (Data curation, Writing—review & editing), Yang Yan (Data curation, Writing—review & editing), Mi Zhou (Data curation, Writing—review & editing), Qiang Zhao (Data curation, Writing—review & editing), Xiaoning Sun (Data curation, Writing—review & editing), Chunsheng Wang (Data curation, Writing—review & editing), Zhenwei Ge (Data curation, Writing—review & editing), Aiqiang Dong (Data curation, Writing—review & editing), and Shengshou Hu (Conceptualization, Supervision, Writing—review & editing)

## Supplementary Material

ezaf365_Supplementary_Data

## Data Availability

The data underlying this article will be shared upon reasonable request to the corresponding author.

## References

[1] Shahim B , Kapelios CJ, Savarese G, Lund LH. Global public health burden of heart failure: an updated review. Card Fail Rev. 2023;9:e11. 37547123 10.15420/cfr.2023.05 PMC10398425

[2] Hieda M , Sata M, Nakatani T. The importance of the management of infectious complications for patients with left ventricular assist device. Healthcare (Basel). 2015;3:750-756. 27417794 10.3390/healthcare3030750 PMC4939583

[3] Kranzl M , Stoiber M, Schaefer AK, et al Driveline features as risk factor for infection in left ventricular assist devices: meta-analysis and experimental tests. Front Cardiovasc Med. 2021;8:784208. 34977190 10.3389/fcvm.2021.784208 PMC8716483

[4] Schloeglhofer T , Stoiber M, Schachl J, et al Mechanical characteristics and infection risks: experimental insights and clinical outcome prediction on left ventricular assist device driveline features. J Heart Lung Transplant. 2025;44:S531.

[5] Wang X , Zhou X, Chen H, et al Long-term outcomes of a novel fully magnetically levitated ventricular assist device for the treatment of advanced heart failure in China. J Heart Lung Transplant. 2024;43:1806-1815. 38740078 10.1016/j.healun.2024.05.004

[6] Zhang J , Chen Z, Griffith BP, Wu ZJ. Computational characterization of flow and blood damage potential of the new maglev CH-VAD pump versus the HVAD and HeartMate II pumps. Int J Artif Organs. 2020;43:653-662. 32043405 10.1177/0391398820903734 PMC11549969

[7] Saeed D , Feldman D, Banayosy AE, et al The 2023 international society for heart and lung transplantation guidelines for mechanical circulatory support: a 10-year update. J Heart Lung Transplant. 2023;42:e1-e222. 10.1016/j.healun.2022.12.004 37245143

[8] Patel CB , Blue L, Cagliostro B, et al Left ventricular assist systems and infection-related outcomes: a comprehensive analysis of the MOMENTUM 3 trial. J Heart Lung Transplant. 2020;39:774-781. 32276809 10.1016/j.healun.2020.03.002

[9] Kormos RL , Antonides CFJ, Goldstein DJ, et al Updated definitions of adverse events for trials and registries of mechanical circulatory support: a consensus statement of the mechanical circulatory support academic research consortium. J Heart Lung Transplant. 2020;39:735-750. 32386998 10.1016/j.healun.2020.03.010

[10] Kusne S , Mooney M, Danziger-Isakov L, et al An ISHLT consensus document for prevention and management strategies for mechanical circulatory support infection. J Heart Lung Transplant. 2017;36:1137-1153. 28781010 10.1016/j.healun.2017.06.007

[11] Bernhardt AM , Schlöglhofer T, Lauenroth V, et al; Driveline Expert STagINg and carE DESTINE Study Group, a Ventricular Assist Device Driveline Infection Study Group. Prevention and early treatment of driveline infections in ventricular assist device patients - the DESTINE staging proposal and the first standard of care protocol. J Crit Care. 2020;56:106-112. 31896443 10.1016/j.jcrc.2019.12.014

[12] Shah P , Sayer G, Sinha SS, et al Dynamic risk estimation of adverse events in ambulatory LVAD patients: a MOMENTUM 3 analysis. JACC Heart Fail. 2024;12:1898-1912. 39066758 10.1016/j.jchf.2024.05.018

[13] Krzelj K , Petricevic M, Gasparovic H, Biocina B, McGiffin D. Ventricular assist device driveline infections: a systematic review. Thorac Cardiovasc Surg. 2022;70:493-504. 34521143 10.1055/s-0041-1731823

[14] Meyer DM , Nayak A, Wood KL, et al The Society of Thoracic Surgeons Intermacs 2024 annual report: focus on outcomes in younger patients. Ann Thorac Surg. 2025;119:34-58. 39442906 10.1016/j.athoracsur.2024.10.003

[15] Aburjania N , Sherazi S, Tchantchaleishvili V, Alexis JD, Hay CM. Stopping conventional showering decreases Pseudomonas infections in left ventricular assist device patients. Int J Artif Organs. 2017;40:282-285. 28430297 10.5301/ijao.5000590

[16] Dean D , Kallel F, Ewald GA, et al; Jun SSI Registry Investigators. Reduction in driveline infection rates: results from the HeartMate II multicenter driveline silicone skin interface (SSI) registry. J Heart Lung Transplant. 2015;34:781-789. [PMC][10.1016/j.healun.2014.11.021] [25735901] 25735901 10.1016/j.healun.2014.11.021

[17] Cowger JA , Schettle S, Pagani FD, et al Heterogeneity in HeartMate 3 implanting center infection management reveals opportunities for quality improvement and best practice initiatives during left ventricular assist device support. J Heart Lung Transplant. 2025 Jul 28:S1053-2498(25)02147-3. 10.1016/j.healun.2025.07.024 40738195

[18] Imamura T , Murasawa T, Kawasaki H, et al Correlation between driveline features and driveline infection in left ventricular assist device selection. J Artif Organs. 2017;20:34-41. 27448017 10.1007/s10047-016-0923-8

