# Supplementary material for: Low Driveline Infection Rates in Patients With a Novel Fully Magnetically Levitated Ventricular Assist Device
Source: Eur J Cardiothorac Surg. 2025 Oct 25;67(12):ezaf365. doi: 10.1093/ejcts/ezaf365 (PMC12673059; doi:10.1093/ejcts/ezaf365)
Supplement: ezaf365_Supplementary_Data [file ezaf365_supplementary_data.docx]

**Supplementary Data for**

*Low Driveline Infection Rates in Patients with a Novel Fully Magnetically Levitated Ventricular Assist Device*

Xianqiang Wang, Xingtong Zhou, Haibo Chen et al.

1. **Supplementary Tables**

**Table S1 Cox Proportional Hazards Regression Analyses**

|  | **Univariate analyses** | | | **Multivariate analyses** | |  |
| --- | --- | --- | --- | --- | --- | --- |
| Variables | | HR (95% CI) | P | HR (95% CI) | P |  |
| Age | | 0.962 (0.931, 0.995) | 0.023 | 0.976 (0.937-1.017) | 0.243 |  |
| Height | | 1.069 (1.010, 1.132) | 0.022 | 1.023 (0.955-1.097) | 0.511 |  |
| Body mass index | | 1.004 (0.901, 1.119) | 0.936 |  |  |  |
| Body surface area | | 4.637 (0.628, 34.257) | 0.133 |  |  |  |
| Stroke | | 0.929 (0.124, 6.960) | 0.943 |  |  |  |
| Myocardial infarction | | 1.498 (0.579, 3.872) | 0.404 |  |  |  |
| Diabetes | | 1.834 (0.729, 4.618) | 0.198 | 1.727 (0.628-4.749) | 0.290 |  |
| Hypertension | | 1.010 (0.365, 2.790) | 0.985 |  |  |  |
| Ischemic etiology | | 1.884 (0.725, 4.893) | 0.193 |  |  |  |
| INTERMACS 3-5 | | 1.477 (0.590, 3.701) | 0.405 |  |  |  |
| LVEF > 20% | | 1.757 (0.582, 5.302) | 0.318 |  |  |  |
| Creatinine | | 0.995 (0.983, 1.007) | 0.413 |  |  |  |
| eGFR | | 1.011 (0.997, 1.025) | 0.134 |  |  |  |
| BUN | | 0.951(0.835, 1.084) | 0.952 |  |  |  |
| Lateral thoracotomy | | 0.313 (0.041, 2.359) | 0.259 |  |  |  |
| CPB time | | 1.002 (0.994, 1.010) | 0.641 |  |  |  |
| Frequent dressing change | | | 5.239 (1.780, 15.424) | 0.003 | 3.773 (1.210-11.762) | 0.022 |
| Driveline exit in right | | | 0.678 (0.248, 1.849) | 0.447 |  |  |
| Internal fixation | | | 0.803 (0.260, 2.483) | 0.703 |  |  |
| External fixation | | | 0.678 (0.248, 1.849) | 0.447 |  |  |
| External velour | | 1.586 (0.613, 4.100) | 0.342 |  |  |  |
| Chlorhexidine disinfection | | 1.816 (0.591, 5.576) | 0.297 |  |  |  |

CI: confidence interval; CPB: cardiopulmonary bypass; HR: hazard ratio; INTERMACS: Interagency Registry for Mechanically Assisted Circulatory Support; LVEF: left ventricular ejection fraction

**Table S2 Cox Competing Hazards Regression Analyses**

|  | Multivariate analyses | |
| --- | --- | --- |
| Variables | HR (95% CI) | P |
| Age | 0.977 (0.929-1.028) | 0.371 |
| Height | 1.026 (0.955-1.103) | 0.483 |
| Diabetes | 1.722 (0.638-4.650) | 0.284 |
| Frequent dressing change | 3.920 (3.286-4.554) | 0.031 |

CI: confidence interval; HR: hazard ratio; LVEF: left ventricular ejection fraction

1. **Supplementary Figures**


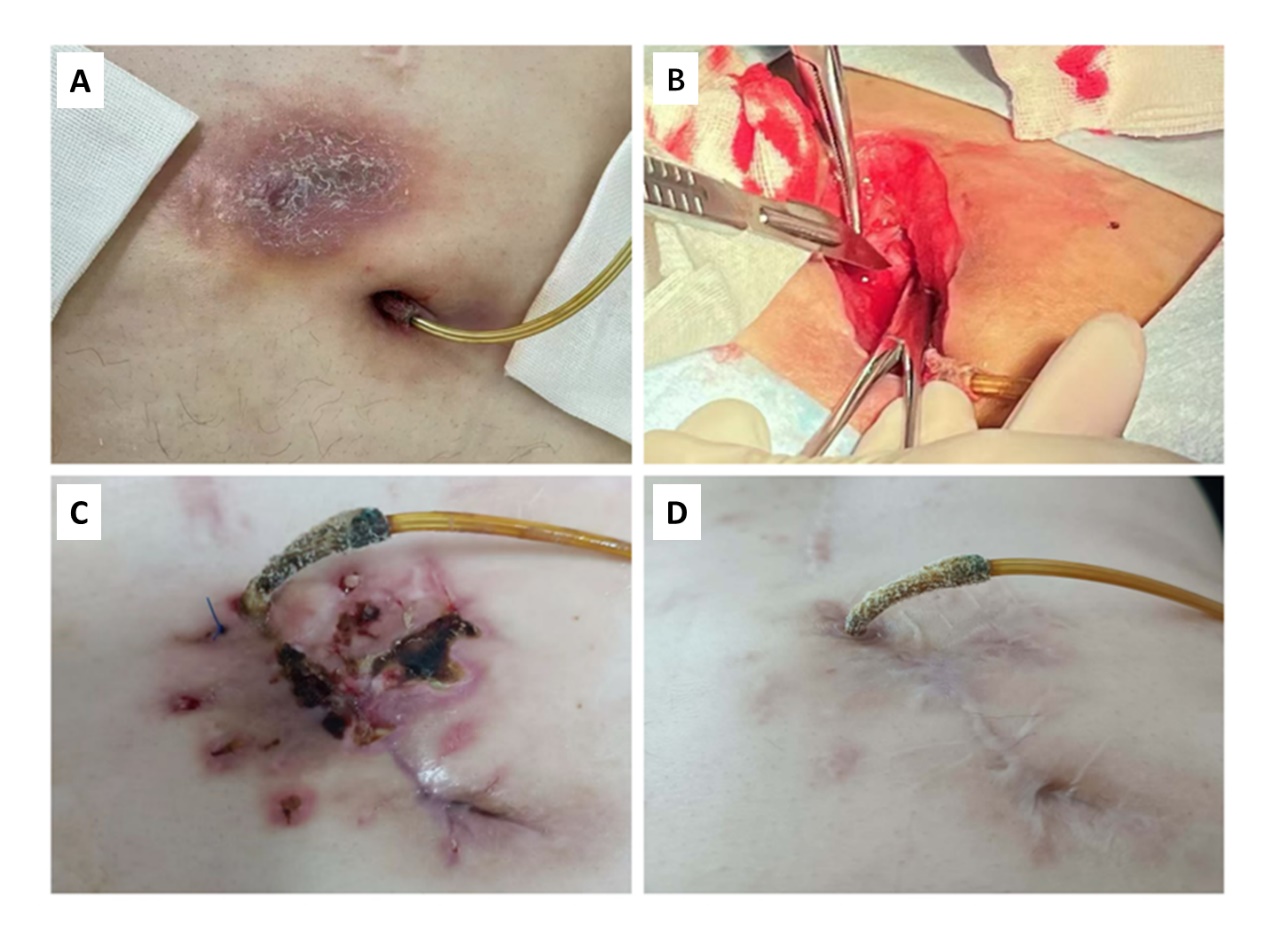
**Figure S1 (A)** Deep driveline infection at 9 months post-implantation. **(B, C)** Driveline relocation surgery was performed at 13 months post-implantation. **(D)** Well-healed exit site at 5.4 years post-implantation with on-going device support.


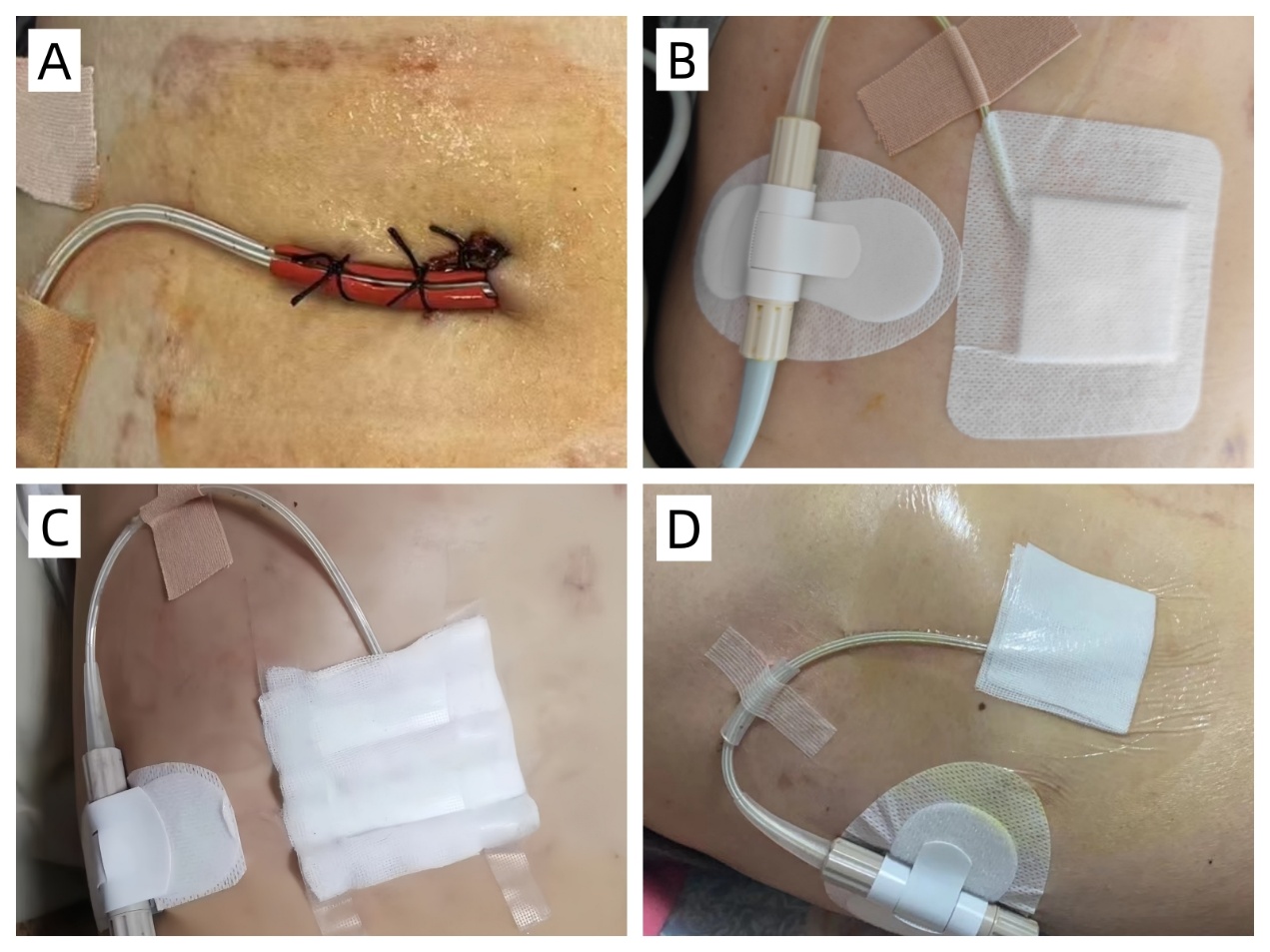


**Figure S2：(A)**Temporary external anchoring sutures were placed in 51% of patients. **(B-D)** Patients used different dressing methods to secure the driveline. The driveline exit sites were covered with non-woven sterile dressings, gauze pads, or transparent waterproof dressings. For driveline fixation, catheter stabilization devices or adhesive tapes were used to maintain immobilization.


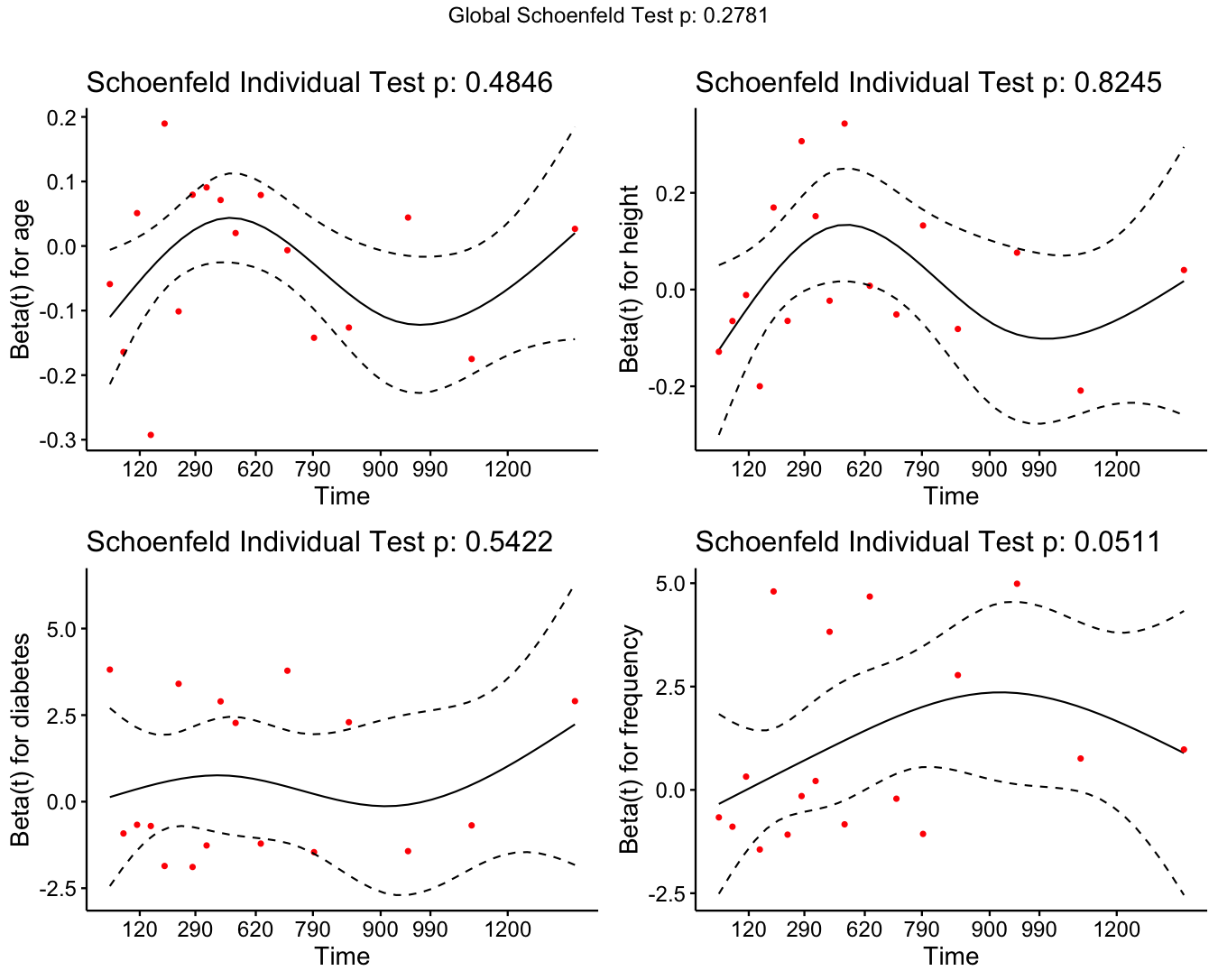


**Figure S3：**Schoenfeld residual plots for proportional hazards assumption testing
